# Risk Factors Associated with Mental Health Outcomes during the Post-Quarantine Period of the COVID-19 in Saudi Population: A Cross-Sectional Study

**DOI:** 10.3390/bs12100391

**Published:** 2022-10-14

**Authors:** Soukaina Ennaceur

**Affiliations:** Department of Public Health, College of Health Sciences, Saudi Electronic University, Jeddah 11673, Saudi Arabia; s.ennaceur@seu.edu.sa; Tel.: +966-542826599

**Keywords:** mental health, sleep quality, post-quarantine, COVID-19, Saudi Arabia

## Abstract

Background: The present study aims to evaluate the mental health symptoms in the Saudi population during the COVID-19 post-quarantine period and to identify the risk factors associated with the severity of the symptoms. Methods: Anxiety was measured with the 7-item Generalized Anxiety Disorder questionnaire, depression with the 9-item Patient Health Questionnaire, insomnia with the 7-item Insomnia Severity Index, and distress with the 22-item Impact Event Scale-Revised questionnaire. Results: A total of 885 respondents answered the online questionnaires. The majority were women (72.8%), married (67.4%), have children (59.3%), and with high education levels (93.2%). The results showed that a high number of the respondents experienced mild to severe symptoms of anxiety (533; 60.3%), depression (659; 47.5%), insomnia (510; 57.6%), and distress (645; 72.9%). The multivariable logistic analysis demonstrated severe anxiety and insomnia among women (OR = 1.71; 95% CI 1.07–1.98; *p* < 0.001 and OR = 2.00; 95% CI 1.78–2.35; *p* = 0.002); severe depression among those under 35 (OR = 2.06; 95% CI 1.97–2.44; *p* = 0.001; and severe distress among non-Saudi respondents (OR = 1.71; 95% CI 1.09–1.93; *p* < 0.001). Conclusions: The results might help in establishing precautionary measures for protecting the mental health of the general population during pandemics.

## 1. Introduction

The first cases of novel coronavirus infection, COVID-19, were reported in the city of Wuhan in China in December 2019 [1]. COVID-19 has caused a global health issue. The World Health Organization [2] declared during its emergency meeting on 30 January 2020, that COVID-19 is a public health emergency of international concern. As of 28 August 2020, the WHO has announced more than 24.2 million confirmed cases of COVID-19, including 827 K deaths globally [3]. In Saudi Arabia, the first case was declared on 2 March 2020. Since that date, the disease has claimed more than 3.7 K lives and affected more than 311 K persons in the country [4].

The severe acute respiratory syndrome coronavirus 2 (SARS-CoV-2) has been identified as a beta-corona virus that affects human health by intermediate transmission through hosts (bats) [5]. Although the transmission routes of the SARS-CoV-2 virus are still under examination, many transmission ways have been discussed, such as transmission through respiratory droplets, aerosols, contaminated surfaces, and fecal-oral routes. The transmission by respiratory droplets was associated with high productivity of the virus in the upper and lower respiratory tract due to direct contact with the contaminated person showing an active cough [6,7,8]. The production of experimental aerosols (similar to those produced by humans) carryingSARS-COV-2 demonstrated the airborne transmission of the virus [9]. Consequently, the deposition of aerosols carrying the virus might cause the contamination of objects and surfaces and infection in humans [10,11]. Another important route of virus spread was reported to be through fecal-oral transmission since RNA-laden aerosols were found on toilet bowls, and SARS-CoV-2 RNA was detected in rectal swabs during the pandemic [10,12,13].The most vulnerable persons to the infection are old adults with medical comorbidities [14]. The WHO had fixed the fatality rate of COVID-19 at around 2%; however, this rate has been estimated to be less than 2% (0.3–0.6%) by some researchers [15].

The quick and dramatic expansion of the disease has resulted in worldwide concern due to both the physical and mental health impacts. Increasing attention is being given by authorities and specialists to understanding how the changes to everyday life and activities might affect people’s mental health and psychological well-being.

Home confinement and lockdown were declared in most countries worldwide to limit the spread of the disease. Psychological consequences were studied to assess the attitude and behaviors of confined people. High prevalence of anxiety, stress, and depression were registered among the general population [16,17] due to the lockdown policies. Children and adolescents are more vulnerable to social distancing and home confinement. It has been shown that students, more specifically, experienced high levels of emotional difficulties related to fear of academic success, task overload, and restriction in pleasant social contact [18,19].

Similar to other countries worldwide, Saudi Arabia introduced on 3 March 2020, a general lockdown and social distancing to minimize the person-to-person transmission of the virus and to stop the spread of the disease. The limitation of everyday life activities such as sports, work, travel, family meetings, and gatherings has seriously impacted the psychological well-being of people. Self-isolation and social distancing have been associated with different feelings of anxiety, depression, guilt, and distress [20].

Previous studies have reported negative psychological responses to the COVID-19 pandemic among the general population during the early stages of the pandemic or the self-isolation and/or quarantine period [21,22,23].

Currently, there are no clear and published data on the psychological impacts of COVID-19 on the general population during the post-quarantine period. Moreover, in Saudi Arabia, no published studies are reporting the association between mental health outcomes and risk factors during the pandemic. The present study was conducted during the first two weeks following the quarantine period of COVID-19 in Saudi Arabia to assess the severity of the symptoms of anxiety, depression, insomnia, and distress among the general population and to identify probable risk factors contributing to mental health disorders in respondents. 

## 2. Material and Methods

### 2.1. Study Design

As the Saudi Government implemented social distancing regulations, respondents were electronically asked to participate in the study. Before this, all questionnaire respondents provided informed consent forms confirming their voluntary participation. The questionnaires were distributed via e-mail and social media (Facebook, Twitter, and WhatsApp). Respondents were informed that they could fill out the questionnaires at any time and without any obligations.

This study is a cross-sectional, community-based survey conducted between 7 July 2020, and 17 July 2020. Data collection was performed using a snowball sampling methodology focusing on the general population from different cities in Saudi Arabia during the COVID-19 pandemic. The questionnaires were sent to university staff and students, their relatives and friends, and they were then asked to share them with their contacts. 

### 2.2. Study Participants

Initially, 1378 respondents agreed to participate however, only 885 completed the questionnaires. Any questionnaire with missing information or incomplete answers was discarded. The first part of the questionnaire focused on the socio-demographic data, where respondents were asked to answer questions about age, sex, educational level, occupation, marital status, number of children, household size, place of residence, and household income.

### 2.3. Study Instruments

Four reviewed and approved questionnaires were used to assess and determine the severity of the mental health symptoms of the general population during the post-quarantine period in Saudi Arabia. The 9-item Patient Health Questionnaire (PHQ-9) [24] was used to assess and measure the severity of depression. The questionnaire used a 4-point Likert scale (0–3) containing 9 items and a total score that ranges from 0 to 27 with cutoff points of 5, 10, 15, and 20 representing mild, moderate, moderately severe, and severe levels of depression [25].

The 7-item Generalized Anxiety Disorder (GAD-7) questionnaire is a 4-point Likert scale (0–3) containing 7 items with a total score ranging from 0 to 21 used to assess the severity of anxiety. Scores of 5, 10, and 15 are considered cutoff points for mild, moderate, and severe anxiety, respectively [26].

The 7-item Insomnia Severity Index (ISI) questionnaire is a 5-point Likert scale used to measure the severity of insomnia. Responses can range from 0 to 4 and the total score ranges from 0–28. A higher score indicates acute insomnia symptoms. Cutoff points were not validated, but total scores can be classified into different categories: a score from 0 to 7 indicates “no significant clinical insomnia”, a score from 8 to 14 indicates “sub-threshold insomnia”, a score from 15 to 21 means “clinical insomnia” (moderate severity) and a score from 22 to 28 means “clinical insomnia” (severe) [27].

The 22-item Impact Event Scale-Revised (IES-R) questionnaire is used to evaluate the distress that people sometimes suffer following a stressful event. The IES-R questionnaire uses a 5-point Likert scale (0–4) with 22 items and a total score ranging from 0 to 88. The questionnaire is divided into three subscales according to the nature of the questions: the intrusion scale, the avoidance scale, and the hyperarousal scale. High scores (over 24) belong to individuals who have been identified to have meaningful distress levels: a score of 24 or more indicates that the post-traumatic stress disorder is considered a clinical concern, and a score of 33 and above is considered the cutoff for a probable diagnosis of posttraumatic stress disorder and a score of 37 and above indicates a severe effect on an individual’s immune system functions (even 10 years after a stressful event) [28].

### 2.4. Data Analysis

The data were analyzed using the SPSS (V. 27.0)(IBM Corporation, Armonk, New York, NY, USA) software for Windows. Since the calculated total scores of the measuring instruments did not follow the normal distribution model, they are presented as medians with interquartile ranges (IQRs). The ranked and counted data related to the symptom severity for each mental health outcome evaluated are presented as numbers and percentages. To compare the severity of each mental health outcome for the different groups, we applied the Mann-Whitney *U* test and the Kruskal-Wallis test. Different risk factors were studied to understand their association with depression, anxiety, distress, and insomnia severity during the post-quarantine period of the COVID-19 pandemic. The study of these associations was performed using multivariable logistic regression analysis. These associations were presented as an odds ratio (OR) and a 95% confidence interval (95% CI). All confounders related to the risk factors were adjusted. All the applied tests were 2-tailed tests with a *p*-value of less than 0.05 for statistical significance.

### 2.5. Ethical Statement

This study was approved by the Ethical Community of Saudi Electronic University (SEUREC-CHS20101).

## 3. Results 

### 3.1. Demographic Characteristics

The socio-demographic characteristics of the study respondents are presented in Table 1. Among the 1378 respondents who agreed to participate in the study, only 885 completed the questionnaires, with a participation rate of 64.2%. The average participant age was 35.26 years (±8.64); 72.8% were female and 27.1% were male. Married respondents constituted 67.4% (*n* = 597), while unmarried respondents (single, divorced and widowed) comprised the remaining 32.5% (*n* = 288). The number of respondents with children was 525 (59.3%), and 59.3% (*n* = 525) of them live in large families (between 3 and 5 members/family). The majority of the respondents were non-Saudi (*n* = 597) and live in urban areas (97.9%). The working respondents represented 66.4% (*n* = 588) of the total, and the non-working (unemployed, retired and students) were only 33.5%. The monthly household incomes of the respondents varied greatly: 11.8% have monthly incomes of more than 20,000 SR, 40.3% have a salary ranging between 10,000 SR and 20,000 SR and the remaining respondents have monthly incomes below 5000 SR. Of all the respondents, 4.7% reported having suffered COVID-19.

### 3.2. Severity of Symptoms in the General Population and among Sub-Groups

The results showed that a high percentage of the population studied exhibits anxiety symptoms (533; 60.3%), depression (659; 74.5%), insomnia (510; 57.6%) and distress (645; 72.9%) (Table 2). It should be noted that some sub-groups show high levels of mental health disorders compared to other sub-groups from the same category.

For example, regarding anxiety symptoms, women (416; 47%), those under 35 (321; 36.3%), married people (365; 41.2%), those with children (318; 35.9%), respondents living in a large family (468; 52.8%) and people with a low monthly income (279; 31.5%) experienced mild to severe symptoms compared to the other respondents from the same category. Moreover, people that had not suffered COVID-19 showed mild to severe symptoms of anxiety (520; 58.7%), depression (579; 65.5%), insomnia (453; 51.1%), and distress (546; 61.8%) compared to those groups that had already suffered COVID-19. The results also show that non-Saudi respondents have severe symptoms of anxiety (356; 40.2%), depression (410; 46.3%), insomnia (357; 40.4%) and distress (423; 47.8%) compared to Saudi respondents. 

The comparison among sub-groups shows severe anxiety symptoms among female respondents (103; 11.6%) compared to their male counterparts (26; 2.9%) with a *p* < 0.001; severe depression symptoms among young respondents (83; 9.8%)compared to old respondents(38; 4.3%) with a *p* < 0.001; severe insomnia symptoms among respondents living in large families (72; 8.8%)compared to respondents living in small families (6; 0.7%) with a *p* < 0.001; and severe distress symptoms among those respondents who have not been infected with COVID-19 (150; 16.9%)compared to those infected (9; 1%) with *p* < 0.001.

The place of residence and the technical title of the respondents were not associated with any symptoms of anxiety, depression, insomnia, and distress. 

### 3.3. Total Score Measurements and Related Factors

Table 3 shows the total scores calculated for the studied mental health outcomes among the general population and the sub-groups. Since the results were not normally distributed, the calculated total scores are presented as a median (±IQR). The median of the total scores for anxiety, depression, insomnia, and distress were 6 (2–10), 8 (4–13), 9 (4–16), and 24 (7.5–39), respectively. The comparison of the total scores within the sub-groups shows a similar trend to the severity of the symptoms.

The scores obtained for GAD-7, PHQ-9, ISI, and IES-R were high for women, people under 35, married respondents with children, respondents with low household monthly incomes, and those who are unemployed. Moreover, respondents that are temporarily residing in KSA (non-Saudi) showed high scores for the four measurements studied. 

The comparison between the different groups aims to identify different probable risk factors associated with severe mental health disorders. In general, significant differences were found between all the studied parameters and severe symptoms of anxiety, depression, insomnia, and distress were registered in the population during the post-quarantine period related to the COVID-19 pandemic (*p* < 0.001). For example, our findings show statistically significant differences between males and females in terms of the GAD-7 median scores for anxiety (4 [2–7.5] vs. 7 [2–12]; *p* < 0.001); the PHQ-9 median scores for depression (6 [3–10] vs. 9 [5–14]; *p* < 0.001); the ISI median scores for insomnia (8.5 [2–13] vs. 10 [5–16]; *p* < 0.001) and the IES-R median scores for distress (15 [4–29] vs. 25 [4–10]; *p* < 0.001). 

The only scores that were found to be equal were those for severe symptoms of anxiety and depression within the family size parameter group. In fact, people living in families with a high number of members show similar levels of anxiety and depression as those living in families with few members. However, the former show higher levels of distress and insomnia when compared to families with fewer members.

### 3.4. Risk Factors Associated with Mental Health Outcomes

The identification of risk factors associated with the high severity of different mental health outcomes in the study respondents was conducted using multivariable logistic regression analysis. All confounding factors were controlled before the analysis of the data. The results show that women, married respondents with children, and young respondents (under 35) experienced severe symptoms of anxiety, depression, insomnia, and distress. Moreover, respondents with a low monthly income and those who are unemployed, and people from countries other than Saudi Arabia showed a higher severity of the different mental health outcomes. For example, severe anxiety and insomnia among women (OR = 1.71; 95% CI 1.07–1.98; *p* < 0.001 and OR = 2.00; 95% CI 1.78–2.35; *p* = 0.002); severe depression among respondents under 35 (OR = 2.06; 95% CI 1.97–2.44; *p* = 0.001; and severe distress among non-Saudi respondents (OR = 1.71; 95% CI 1.09–1.93; *p* < 0.001) (Table 4).

The pair-wise comparison revealed high severity symptoms for example in secondary educated respondents when compared to university educated respondents (anxiety: OR = 1.50; 95% CI 1.01–1.96; *p* = 0.005; depression: OR = 1.74; 95% CI 1.25–1.98; *p* = 0.0025; insomnia: OR = 1.36; 95% CI 1.06–1.98; *p* = 0.008 and distress: OR = 0.93; 95% CI 0.57–1.45; *p* = 0.040). It has also been found that family size is an important risk factor that affects the severity of the different targeted mental health outcomes during stressful events (anxiety: OR = 1.79; 95% CI 1.43–1.92; *p* = 0.002; depression: OR = 1.63; 95% CI 1.49–1.896; *p* = 0.001; insomnia: OR = 1.69; 95% CI 1.11–1.84; *p* = 0.001 and distress: OR = 1.68; 95% CI 1.49–1.896; *p* = 0.001).

## 4. Discussion

Our findings show that during the early stages of the post-quarantine period due to COVID-19, a considerable number of people from the Saudi population experienced moderate to severe symptoms of anxiety (80; 9%), depression (55; 6.2%), insomnia (89; 10.5%), and distress (129; 14.6%). The prevalence of the moderate to severe symptoms of distress as measured using the IES-R was higher than the prevalence of the symptoms of anxiety, depression, and insomnia measured by the GAD-7, the PHQ-9, and the ISI, respectively. Similar results were registered in a study conducted in Spain, where the mental problems in the general population tripled compared to the post-confinement period. The prevalence of depression (22.8%), anxiety (26.9%), and lack of mental well-being (75%) were higher during the six to ten weeks of confinement when compared to the before confinement period [17].

Respondents were divided into different sub-groups according to their socio-demographic characteristics to study group differences. The majority of the respondents were female, young people under 35, married with children, non-Saudi residents, employed and with a university education, people living in large families, and people who had not been infected by COVID-19. Our study shows that women experienced greater psychological effects during the COVID-19 pandemic expressed as moderate to severe symptoms of anxiety, depression, insomnia, and distress. This result is in accordance with different studies regarding the psychological impacts of COVID-19 on the general population. A study conducted in Australia showed that women had significantly higher stress scores compared to males (mean ± SD: 5.5 (±4.7) vs.4.9 (±5.1); *p* = 0.005) during the COVID-19 outbreak [21]. Moreover, a Spanish meta-analysis showed that women were among the groups at greater risk of mental ill-health. They showed worries about themselves or their families being contaminated by the SARS-CoV-2 [17].

Younger people (under 35 years) suffered important mental health symptoms and high levels of anxiety, depression, insomnia, and distress. Similar trends were observed in a study conducted in China, where authors demonstrated that the prevalence of anxiety and depressive symptoms were significantly higher in respondents younger than 35 years than in respondents aged 35 years or older (*p* < 0.001) [22]. In addition, another study demonstrated that more younger participants (ages 15–24 years) experienced higher mental health problems than other age groups: 42.5% for depression, 37.3%for anxiety, and 91% for lack of mental well-being [17].

Our results demonstrate that secondary educated respondents show high symptoms of anxiety, depression, insomnia, and distress when compared to well-educated (university-level) respondents. These findings could be explained by the fact that respondents with a high educational level obtained accurate and up-to-date information from official sources (WHO website, MOH, etc.). The content of the health information, based on evidence, could explain the association between the low psychological impacts on those people with higher education compared to low educated respondents still in secondary. An Australian study demonstrated a significant negative association between years of education and psychological disorder, mainly for depression [21]. Similarly, a cross-sectional web-based population conducted in Catalonia five weeks after imposed confinement found that participants with a primary level of education were among the most affected groups by high levels of mental ill-health [17]. 

Married respondents with children living in small families were at high risk of experiencing moderate to severe symptoms of psychological disorders. This might be related to the fact that the majority of the respondents belonging to these sub-groups were worried about their family members, especially children. Similar results were shown by a study conducted in China, demonstrating that about 75.2% of respondents were very worried or somewhat worried about other family members getting COVID-19, and 50.9% of respondents were very worried or somewhat worried about a child younger than 16 years getting the COVID-19 [23]. Moreover, social distancing and self-isolation adopted to limit the extension of the infection negatively influenced the mental health of the respondents. Social support from family is demonstrated to be associated with low levels of mental health disorders, mainly during stressful events [29,30], which could explain the lower psychological impact that the measures taken had on people living in larger families found in the present study. This result was in agreement with a previous study that showed perceived support from family members made unique contributions to their attitudes about social distancing and positive mental health both directly and indirectly (via buffering loneliness) [31].

The present study shows that unemployed respondents and respondents with low monthly incomes experience worse mental health outcomes for the four dimensions considered. Indeed, many people were forced to leave their jobs during the lockdown imposed by the government during the COVID-19 pandemic. This situation caused stressful conditions for people with temporary jobs, people with low monthly incomes, and the unemployed. In similar studies, authors found that worrying about job loss is associated with more than one and a half times the likelihood of mental ill-health [17]. Also, participants from the lowest monthly income category had significantly higher depression levels compared to higher monthly income categories [21].

Our results suggest that participants who have not been infected with COVID-19 experienced severe symptoms of distress during the confinement period more than those already infected by the disease. These findings are similar to review studies [32] suggesting that uninfected people, mainly families, and friends of COVID-19 patients, were more susceptible to psychological disorders because they are worried about infecting themselves and being quarantined. 

The comparison between native Saudi respondents and non-Saudi residents revealed significant differences in terms of the severity of the symptoms for all measurements. The psychological responses of non-Saudi residents to COVID-19 may have different causes, including the feeling of loneliness and vulnerability, worries about health control and concerns about their family health, changes to working conditions, and social distancing and self-isolation. Furthermore, a previous study conducted in Saudi Arabia found that infected people with COVID-19 have experienced high levels of depression when compared to Saudis [33].

Since the COVID-19 epidemic has spread worldwide and has infected the psychological well-being of the general population, our results could have great implications for the health system and policies. Our study has identified the sub-groups at high risk of developing mental health disorders based on their socio-demographic characteristics. Public health authorities would be interested in our findings to establish the appropriate intervention strategies for early protection programs. Moreover, our findings support the need to modify and improve psychological interventions during a crisis to meet the needs of the general population. Health authorities and policymakers could use our data to enhance methods of psychological well-being support, such as providing online support and behavior therapy to counteract anxiety, depression, and insomnia in a confined environment. Based on our findings, further investigation should be conducted to identify the general population’s needs for mental health support during an epidemic and to evaluate the existing intervention strategies established to protect the general population during the COVID-19 pandemic

The present study was conducted during the first two weeks following the COVID-19 pandemic quarantine period and has some limitations, such as the lack of longitudinal follow-up, given that the study was conducted for ten days. Even though the participation rate was 64.2%, the respondents who did not complete the questionnaires or who refused to participate may not have been interested in our study or may have not suffered any stress at all during this period. Due to social distancing, the data collection was carried out using a snowball strategy which enabled us to extrapolate the results to the whole population, given its lack of randomization.

However, our study has a number of strengths. Firstly, this is, to the best of our knowledge, the first published investigation conducted to assess the mental well-being of the Saudi population outcomes during the post-quarantine period of COVID-19. Secondly, the findings of the present study can be used as a reference by health authorities and professionals to establish well-structured psychological and mental health plans for mental health protection and support among the Saudi population during stressful events. Finally, the findings of the present study have helped in the identification of groups from the general population at a high risk of suffering mental health disorders during pandemics based on the socio-demographic data of the respondents.

## 5. Conclusions

During the post-quarantine period of COVID-19, the prevalence of mental health symptoms: anxiety, depression, insomnia, and distress was higher for respondents with some demographic characteristics in the studied sample such as women, young participants, married people, those with children, respondents living in large families, people with low monthly income, people that had not suffered COVID-19 and non-Saudi respondents. 

Data from the present study highlighted sub-group at risk during infection and who require mental well-being services to support them during this stressful period. Our findings could be useful for governmental and private agencies concerned with the mental health of the general population during crises to establish well-studied mental health promotion programs and policies that target groups at high risk of mental health problems. 

## Figures and Tables

**Table 1:** Sociodemographic characteristics of the study respondents.

Characteristics		Number	Percentage (%)
	Male	240	27.1
	Female	645	72.8
Age	≤25	114	12.9
	26–35	339	38.3
	36–45	342	38.6
	≥46	90	10.1
Marital status	Married	597	64.4
	Unmarried ^1^	288	32.5%
Number of children	All of them are 15 or older	42	4.7
	One or more is/are under 15	483	54.5
	None	360	40.6
Household size	One person	33	3.7
	Two persons	96	10.8
	3–5 persons	525	59.3
	≥6 persons	231	26.1
Nationality	Saudi	318	35.9
	Non-Saudi	567	64.0
Residence area	Urban	867	97.9
	Rural	18	2.0
Education	Primary school	0	0.0
	Secondary school	60	6.7
	University-Bachelor’s	396	44.7
	University-Master’s	189	21.3
	University-PhD	240	27.1
Occupation	Employed	588	66.4
	Unemployed	129	14.5
	Retired	12	1.3
	Student	156	17.6
Household income/month	<5000 SR	192	21.6
	5000–10,000 SR	231	26.1
	10,000–20,000 SR	357	40.3
	>20,000 SR	105	11.8
Suffered COVID-19	Yes	42	4.7
	No	843	95.2

^1^: single, divorced and widowed.

**Table 2:** Severity of anxiety, depression, insomnia and distress symptoms in the general population and among different groups.

**Severity of Symptoms**	**Total** **N (%)**	**Gender**		***p*-Value**	**Age**		***p*-Value**	**Marital Status**		***p*-Value**	**Children**		***p*-Value**	**Family Size**		***p*-Value**
		**Male**	**Female**		**<35**	**≥35**		**Married**	**Unmarried**		**Yes**	**No**		**<3**	**≥3**	
**GAD-7 foranxiety**																
Minimal 0–4	352 (39.8)	120 (13.6)	229 (25.9)	<0.001	132 (14.9)	190 (21.5)	<0.001	232 (26.2)	117 (13.2)	0.002	207 (23.4)	138 (15.6)	0.040	50 (5.6)	288 (32.5)	0.006
Mild 5–9	276 (31.2)	68 (7.7)	205 (23.2)		147 (16.6)	99 (11.2)		179 (20.2)	97 (11.0)		165 (18.6)	114 (12.9)		41 (4.6)	240 (27.1)	
Moderate 10–14	136 (15.4)	26 (2.9)	108 (12.2)		113 (12.8)	73 (8.2)		98 (11.1)	47 (5.3)		72 (8.1)	67 (7.6)		20 (2.3)	117 (13.2)	
Severe 15–21	121 (13.7)	26 (2.9)	103 (11.6)		61 (6.9)	70 (7.9)		88 (9.9)	27 (3.1)		81(9.2)	41 (4.6)		18 (2.0)	111 (12.5)	
**PHQ-9 for depression**																
Minimal 0–4	226 (25.5)	73 (8.2)	138 (15.6)	0.001	76 (8.6)	89 (10.1)	0.001	152 (17.2)	70 (7.9)	<0.001	135 (15.3)	86 (9.7)	0.001	39 (4.4)	177 (20.0)	0.009
Mild 5–9	305 (34.5)	85 (9.6)	220 (24.9)		141 (15.9)	164 (18.5)		205 (23.2)	99 (11.2)		189 (21.4)	120 (13.6)		44 (5.0)	267 (30.2)	
Moderate 10–14	202 (22.8)	38 (4.3)	164 (18.5)		105 (11.9)	97 (11.0)		138 (15.6)	64 (7.2)		102 (11.5)	102 (11.5)		38 (4.3)	168 (19.0)	
Moderately severe 15–19	85 (9.6)	14 (1.6)	88 (9.9)		44( 5.0)	44 (5.0)		52 (5.9)	35 (4.0)		57 (6.4)	32 (3.6)		0 (0.0)	90 (10.2)	
Severe 20–27	67 (7.6)	30 (3.4)	35 (4.0)		87 (9.8)	38 (4.3)		50 (5.6)	20 (2.3)		42 (4.7)	20 (2.3)		8 (0.9)	54 (6.1)	
**ISI for insomnia**																
Absence 0–7	375 (42.4)	117 (13.2)	246 (27.8)	<0.001	243 (27.5)	135 (15.3)	0.002	228 (25.8)	108 (12.2)	0.030	198 (22.4)	165 (18.6)	0.050	60 (6.8)	303 (34.2)	<0.001
Subthreshold 8–14	264 (29.8)	78 (8.8)	192 (21.7)		121 (13.7)	158 (17.9)		204 (23.1)	66 (7.5)		183 (20.7)	87 (9.8)		36 (4.1)	234 (26.4)	
Moderate 15–21	170 (19.2)	39 (4.4)	135 (15.3)		77 (8.7)	102 (11.5)		111 (12.5)	51 (5.8)		93 (10.5)	81 (9.2)		27 (3.1)	147 (16.6)	
Severe 22–28	76 (8.6)	6 (0.7)	72 (8.1)		12 (1.4)	37 (4.2)		54 (6.1)	63 (7.1)		51 (5.8)	27 (3.1)		6 (0.7)	72 (8.1)	
**IES-R for distress**																
Normal 0–8	240 (27.1)	93 (10.5)	147 (16.6)	<0.001	156 (17.6)	84 (9.5)	0.030	162 (18.3)	78 (8.8)	0.002	141 (15.9)	99 (11.2)	0.080	30 (3.4)	210 (23.7)	0.050
Mild 9–25	237 (26.8)	60 (6.8)	177 (20.0)		114 (12.9)	123 (13.9)		141 (15.9)	96 (10.8)		126 (14.2)	111 (12.5)		33 (3.7)	204 (23.1)	
Moderate 26–43	249 (28.1)	63 (7.1)	186 (21.0)		111 (12.5)	138 (15.6)		183 (20.7)	66 (7.5)		156 (17.6)	93 (10.5)		42 (4.7)	207 (23.4)	
Severe 44–88	159 (18.0)	24 (2.7)	135 (15.3)		72 (8.1)	87 (9.8)		111 (12.5)	48 (5.4)		102 (11.5)	57 (6.4)		24 (2.7)	135 (15.3)	
**Severity of Symptoms**	**Nationality**			***p*-Value**	**Occupation**		***p*-Value**	**Education**		***p*-Value**	**Monthly Income** **(SR)**		***p*-Value**	**SufferedCOVID-19**		*p*-Value
	**Saudi**	**Non-Saudi**			**Employed**	**Unemployed**		**Secondary**	**University**		**<10,000**	**≥10,000**		**Yes**	**No**	
**GAD-7 foranxiety**																
Minimal 0–4	141 (15.9)	211 (23.8)		<0.001	244 (27.6)	105 (11.9)	0.001	20 (2.3)	329 (37.2)	0.005	144 (16.3)	208 (23.5)	0.020	24 (2.7)	323 (36.5)	0.001
Mild 5–9	97 (11.0)	179 (20.2)			182 (20.6)	94 (10.6)		17 (1.9)	258 (29.2)		149 (16.8)	126 (14.2)		5 (0.6)	270 (30.5)	
Moderate 10–14	47 (5.3)	88 (9.9)			94 (10.6)	41 (4.6)		8 (0.9)	126 (14.2)		70 (7.9)	64 (7.2)		5 (0.6)	132 (14.9)	
Severe 15–21	33 (3.7)	89 (10.1)			68 (7.7)	57 (6.4)		15 (1.7)	112 (12.7)		60 (6.8)	64 (7.2)		8 (0.9)	118 (13.3)	
**PHQ-9 for depression**																
Minimal 0–4	73 (8.2)	157 (17.7)		0.030	166 (18.8)	64 (7.2)	0.010	14 (1.6)	212 (24.0)	0.009	104 (11.8)	125 (14.1)	<0.001	12 (1.4)	217 (24.5)	0.040
Mild 5–9	120 (13.6)	185 (20.9)			202 (22.8)	102 (11.5)		14 (1.6)	291 (32.9)		141 (15.9)	164 (18.5)		14 (1.6)	291 (32.9)	
Moderate 10–14	70 (7.9)	132 (14.9)			135 (15.3)	67 (7.6)		26 (2.9)	176 (19.9)		108 (12.2)	94 (10.6)		8 (0.9)	195 (22.0)	
Moderately severe 15–19	38 (4.3)	49 (5.5)			47 (5.3)	41 (4.6)		2 (0.2)	85 (9.6)		47 (5.3)	41 (4.6)		0 (0.0)	88 (9.9)	
Severe 20–27	17 (1.9)	44 (5.0)			38 (4.3)	23 (2.6)		4 (0.5)	61 (6.9)		23 (2.6)	38 (4.3)		8 (0.9)	52 (5.9)	
**ISI for insomnia**																
Absence 0–7	153 (17.3)	210 (23.7)		0.040	258 (29.2)	114 (12.9)	0.008	24 (2.7)	339 (38.3)	0.004	186 (21.0)	177 (20.0)	0.060	18 (2.0)	345 (39.0)	0.030
Subthreshold 8–14	72 (8.1)	198 (22.4)			183 (20.7)	87 (9.8)		15 (1.7)	255 (28.8)		123 (13.9)	147 (16.6)		12 (1.4)	258 (29.2)	
Moderate 15–21	60 (6.8)	114 (12.9)			102 (11.5)	63 (7.1)		18 (2.0)	156 (17.6)		81 (9.2)	93 (10.5)		3 (0.3)	171 (19.3)	
Severe 22–28	33 (3.7)	45 (5.1)			45 (5.1)	33 (3.7)		3 (0.3)	75 (8.5)		33 (3.7)	45 (5.1)		9 (1.0)	69 (7.8)	
**IES-R for distress**																
Normal 0–8	96 (10.8)	144 (16.3)		0.070	165 (18.6)	75 (8.5)	<0.001	24 (2.7)	216 (24.4)	0.003	111 (12.5)	132 (14.9)	0.020	6 (0.7)	231 (26.1)	<0.001
Mild 9–25	93 (10.5)	144 (16.3)			162 (18.3)	75 (8.5)		9 (1.0)	228 (25.8)		114 (12.9)	123 (13.9)		15 (1.7)	225 (25.4)	
Moderate 26–43	69 (7.8)	180 (20.3)			168 (19.0)	81 (9.2)		15 (1.7)	234 (26.4)		120 (13.6)	129 (14.6)		12 (1.4)	237 (26.8)	
Severe 44–88	60 (6.8)	99 (11.2)			93 (10.5)	66 (7.5)		12 (1.4)	147 (16.6)		78 (8.8)	78 (8.8)		9 (1.0)	150 (16.9)	

**Table 3:** Total scores of the studied health outcomes in the general population and among different groups.

**Scale**	**Total Score: Median (IQR)**	**Gender**		***p*-Value**	**Age**		***p*-Value**	**Marital Status**		***p*-Value**	**Children**		***p*-Value**	**Family Size**		***p*-Value**
		**Male**	**Female**		**<35**	**≥35**		**Married**	**Unmarried**		**No**	**Yes**		**<3**	**≥3**	
GAD-7 for anxiety	6.0 (2–10)	4.0 (2–7.5)	7.0 (2–12)	<0.001	7.0 (3–11)	5.0 (2–10)	<0.001	6.0 (3–10)	6.0 (2–10)	0.009	6.0 (3–10)	7.0 (2–11)	0.007	7.0 (1–9.5)	6.0 (3–10)	0.030
PHQ-9 for depression	8.0 (4–13)	6.0 (3–10)	9.0 (5–14)	<0.001	9 (5–13)	7.0 (4–13)	<0.001	6.0 (3–10)	8.0 (4–13)	0.003	7.0 (4–13)	8.0 (5–13)	<0.001	8.0 (4–12)	8.0 (4–13)	0.090
ISI for insomnia	9.0 (4–16)	8.5 (2–13)	10.0 (5–16)	<0.001	10.0 (6–16)	9.0 (3–15)	0.004	9.0 (3.5–15)	9.0 (5–16)	0.010	9.0 (3.5–15)	9.0 (5–10)	0.008	9.5 (4–16)	8.0 (9–14)	0.060
IES-R for distress	24.0 (7.5–39)	15.0 (4–29)	25.0 (10–45)	<0.001	24.0 (10–42)	22.0 (6–38)	<0.001	24.0 (8–38.5)	20.0 (7–39)	<0.001	22.0 (7–30)	24.0 (8–42)	0.003	24.0 (7–41)	20.0 (9–36)	0.070
**Scale**	**Nationality**		***p*-Value**		**Occupation**		***p*-Value**	**Education**		***p*-Value**	**Monthly Income (SR)**		***p*-Value**	**Suffered COVID-19**		***p*-Value**
	**Saudi**	**Non-Saudi**			**Employed**	**Unemployed**		**Secondary**	**University**		**<10,000**	**≥10,000**		**Yes**	**No**	
GAD-7 for anxiety	5.0 (2–10)	7.0 (3–10)	<0.001		6.0 (2–10)	7.0 (3–12)	0.008	6.0 (3–12)	6.0 (2–10)	0.009	7.0 (3–11)	5.0 (2–10)	<0.001	3,5 (1–10)	6.0 (3–10)	<0.001
PHQ-9 for depression	7.0 (5–13)	8.0 (4–13)	<0.001		7.0 (4–13)	9.0 (5–13.5)	0.001	9.5 (5.5–11.5)	7.0 (4–13)	0.002	9.0 (5–13)	7.0 (4–13)	<0.001	8.0 (4–13)	8.0 (4–13)	0.006
ISI for insomnia	8.0 (5–15.5)	10.0 (3–16)	<0.001		9.0 (3–15)	10.0 (5–16.5)	0.005	11.0 (5.5–16)	9.0 (4–15.5)	0.030	10.0 (4–16)	9.0 (4–15)	<0.001	9.0 (4–7)	9.0 (4–16)	0.040
IES-R for distress	20.0 (6–39)	25.0 (8–39)	<0.001		22.0 (6.5–38)	25.0 (9–42)	<0.001	22.0 (6.5–42)	24.0 (8–39)	0.004	24.0 (8–40)	22.0 (7–39)	<0.001	25.0 (15–36)	23.0 (7–40)	<0.001

**Table 4:** Multivariable Logistic Regression Analysis to assess the probable risk factors of mental health outcomes.

Variables		Gender		Age		Marital Status		Children		Family Size	
		Male	Female	<35	≥35	Married	Unmarried	Yes	No	<3	≥3
**GAD-7 for anxiety**											
Number of severe symptoms/Total (%)		52/240 (21.6)	211/654 (32.2)	174/453 (38.4)	143/432 (33.1)	186/597 (31.1)	74/288 (25.6)	153/525 (29.1)	108/360 (30.0)	38/149 (25.5)	228/756 (30.1)
OR (95% CL)		1 [Ref]	1.72 (1.07–1.98)	1.26 (0.96–1.85)	1 [Ref]	1.30 (0.67–1.88)	1 [Ref]	0.95 (0.51–1.25)	1 [Ref]	1.79 (1.43–2.08)	1 [Ref]
*p* value	By category	NA	<0.001	0.03	NA	<0.001	NA	0.050	NA	0.002	NA
	Overall	<0.001		0.003		<0.001		0.050		0.002	
**PHQ-9 for depression**											
Number of severe symptoms/Total (%)		72/240 (30.0)	287/654 (43.8)	236/453 (52.0)	179/432 (41.4)	240/597 (40.2)	119/288 (41.3)	201/525 (38.2)	154/360 (42.7)	46/149 (30.8)	312/756 (41.2)
OR (95% CL)		1 [Ref]	1.82 (1.37–2.01)	2.06 (1.97–2.44)	1 [Ref]	0.95 (0.67–1.13)	1 [Ref]	0.82 (0.57–1.21)	1 [Ref]	1.63 (1.49–1.96)	1 [Ref]
*p* value	By category	NA	<0.001	0.001	NA	0.006	NA	0.010	NA	0.001	NA
	Overall	<0.001		0.001		0.006		0.010		0.001	
**ISI for insomnia**											
Number of severe symptoms/Total (%)		45/240 (18.7)	207/654 (31.6)	89/453 (19.6)	139/432 (57.4)	165/597 (27.6)	114/288 (39.5)	144/525 (27.4)	108/360 (30.0)	33/149 (22.1)	219/756 (28.9)
OR (95% CL)		1 [Ref]	2.00 (1.78–2.35)	1.94 (1.34–2.12)	1 [Ref]	0.58 (0.35–88)	1 [Ref]	0.88 (0.51–1.21)	1 [Ref]	1,69 (1.49–2.06)	1 [Ref]
*p* value	By category	NA	0.002	0.001	NA	0.090	NA	0.020	NA	0.004	NA
	Overall	0.002		0.001		0.090		0.020		0.004	
**IES-R for distress**											
Number of severe symptoms/Total (%)		87/240 (36.2)	321/654 (49.0)	183/453 (40.3)	225/432 (52.0)	294/597 (50.0)	114/288 (39.5)	258/525 (49.1)	140/360 (38.8)	66/149 (44.2)	242/756 (32.0)
OR (95% CL)		1 [Ref]	1.69 (1.09–1.93)	0.62 (0.56–0.89)	1 [Ref]	1.48 (1.13–1.70)	1 [Ref]	1.51 (1.24–1.96)	1 [Ref]	1.68 (1.11–1.84)	1 [Ref]
*p* value	By category	NA	<0.001	0.08	NA	0.003	NA	0.002	NA	0.001	NA
	Overall	<0.001		0.080		0.003		0.002		0.001	
Variables		Nationality		Occupation		Education		Monthly incomes (SR)		Suffered COVID-19	
		Saudi	Non-Saudi	Employed	Unemployed	Secondary	University	< 10.000	≥ 10.000	Yes	No
**GAD-7 for anxiety**											
Number of severe symptoms/Total (%)		80/318 (25.1)	177/567 (31.2)	162/588 (27.5)	98/297 (32.9)	23/60 (38.3)	238/815 (29.2)	130/423 (30.7)	128/462 (27.7)	13/42 (30.9)	250/843 (29.6)
OR (95% CL)		1 [Ref]	1.35 (1.09–1.57)	1 [Ref]	1.29 (0.89–1.68)	1.50 (1.01–1.96)	1 [Ref]	1.15 (0.87–1.32)	1 [Ref]	1.06 (0.74–1.77)	1 [Ref]
*p* value	By category	NA	0.003	NA	0.002	0.005	NA	<0.001	NA	0.006	NA
	Overall	0.003		0.002		0.005		<0.001		0.006	
**PHQ-9 for depression**											
Number of severe symptoms/Total (%)		125/318 (39.3)	215/567 (37.9)	220/588 (37.4)	131/297 (44.1)	32/60 (53.3)	322/815 (39.5)	178/423 (42.0)	173/462 (37.4)	16/42 (38.0)	344/843 (40.8)
OR (95% CL)		1 [Ref]	0.94 (0.65–1.21)	1 [Ref]	1.32 1.07–1.67)	1.74 (1.25–1.98)	1 [Ref]	1.21 (0.86–1.42)	1 [Ref]	0.89 (61–1.13)	1 [Ref]
*p*-value	By category	NA	0.060	NA	0.004	0.002	NA	0.003	NA	0.050	NA
	Overall	0.060		0.004		0.002		0.003		0.050	
**ISI for insomnia**											
Number of severe symptoms/Total (%)		93/318 (29.2)	159/567 (28.0)	147/588 (25.0)	96/297 (32.3)	21/60 (35.0)	231/815 (28.3)	114/423 (26.9)	138/462 (29.8)	12/42 (28.5)	240/843 (28.4)
OR (95% CL)		1 [Ref]	0.94 (0.75–1.33)	1 [Ref]	1.4 (1,15–1.87)	1.36 (1.06–1.98)	1 [Ref]	1.21 (0.87–1.78)	1 [Ref]	1.00 (0.62–1.65)	1 [Ref]
*p* value	By category	NA	0.020	NA	0.002	0.008	NA	0.002	NA	0.004	NA
	Overall	0.020		0.002		0.008		0.002		0.004	
**IES-R for distress**											
Number of severe symptoms/Total (%)		129/318 (40.5)	279/567 (49.2)	261/588 (44.3)	147/297 (49.4)	27/60 (45.0)	381/815 (46.7)	198/423 (47.8)	207/462 (44.8)	21/42 (50.0)	387/843 (45.9)
OR (95% CL)		1 [Ref]	1.71 (1.23–1.86)	1 [Ref]	1.22 (0.78–1.65)	0.93 (0.57–1.45)	1 [Ref]	0.99 (0.64–1.65)	1 [Ref]	1.17 (0.78–1.67)	1 [Ref]
*p* value	By category	NA	<0.001	NA	0.010	0.040	NA	0.030	NA	<0.001	NA
	Overall	<0.001		0.010		0.040		0.030		<0.001	

## Data Availability

All data generated or analyzed during this study are included in this published article.
